# WOMEN’S HEALTH: PARADOXICAL HEALTH DISPARITIES AMONG ASIAN AMERICAN WOMEN

**DOI:** 10.1093/geroni/igac059.2845

**Published:** 2022-12-20

**Authors:** Janella Camp, Laura Bernstein, Julie Hicks Patrick

**Affiliations:** West Virginia University, Morgantown, West Virginia, United States; West Virginia University, Morgantown, West Virginia, United States; West Virginia University, Morgantown, West Virginia, United States

## Abstract

Asian Americans are among the fastest growing ethnic minority groups in the U.S (Budiman & Ruiz, 2017), but women’s healthcare is understudied. This may allow potential health disparities to go unnoticed. Our study aims to determine whether Asian American women are utilizing preventative health care services and to examine relations with self-reported health. We used data from a national sample of American women (Nf 58,934; mean age = 47.3 years; range 18 to 80+) from the 2020 Behavioral Risk Factor Surveillance System (BRFSS) data of the Centers for Disease Control and Prevention (CDC). We examined the recency of receiving a PAP smear, a mammogram, and the HPV test, along with subjective assessments of health. Asian American women reported less recent PAP smears, mammograms, and HPV tests, relative to their counterparts. However, Asian American women reported better general and physical health than non-Asian American women. To examine whether Asian American status contributed to health reports above and beyond that accounted for by the preventative tests and age, we conducted a 3-step hierarchical regression. Even after controls, Asian American status accounted for unique variance in health outcomes [F (1, 58,928) = 36.51, p < .001]. Post hoc exploratory analyses further examine the role of race in women’s preventative health care. Our findings indicate that Asian American women report less use of medical services, but better general and physical health. These results suggest that further studies are needed to explore other health behaviors that may account for better health reports among Asian American women.

